# Real-world comparison of brain [^18^F]FDG-PET imaging with CSF Alzheimer's disease biomarkers in a tertiary memory clinic setting

**DOI:** 10.1016/j.eclinm.2026.103910

**Published:** 2026-04-20

**Authors:** Neus Rabaneda-Lombarte, João Pedro Ferrari-Souza, Johanna Celedon, Eduardo R. Zimmer, Bradford C. Dickerson, Steven E. Arnold, Pia Kivisäkk, Alberto Serrano-Pozo

**Affiliations:** a Mass General Brigham Neurology Department, MGH Campus, Boston, MA 02114, USA; b Massachusetts Alzheimer's Disease Research Center, Charlestown, MA 02129, USA; c Harvard Medical School, Boston, MA 02115, USA; d Department of Pharmacology, Universidade Federal do Rio Grande do Sul, Porto Alegre, RS, Brazil; e Neuroscience Unit, Hospital Moinhos de Vento, Porto Alegre, RS, Brazil

**Keywords:** Alzheimer's disease, CSF AD biomarkers, [^18^F]FDG-PET imaging

## Abstract

**Background:**

[^18^F]FDG-PET brain scan remains widely used in the evaluation of cognitive decline worldwide, however data on its diagnostic performance against gold-standard CSF Alzheimer's disease (AD) biomarkers are scarce. We aimed to assess the agreement between [^18^F]FDG-PET findings and CSF AD biomarkers in a real-world tertiary memory clinic setting.

**Methods:**

Cross-sectional study of Mass General Brigham patients with cognitive concerns and available [^18^F]FDG-PET imaging and CSF AD biomarkers between 01/01/2013 and 06/30/2025. [^18^F]FDG-PET brain scan findings were categorized as “Normal,” “Abnormal Inconclusive,” “Abnormal Not AD-like,” or “Abnormal AD-like,” based on the narrative report. The CSF AD biomarker panel was classified as “Not AD,” “Equivocal,” or “Consistent with AD” following the lab report. [^18^F]FDG-PET was compared with gold-standard CSF AD biomarkers using kappa agreement test and regression models.

**Findings:**

Among 360 eligible individuals, 151 had a CSF profile “Consistent with AD,” 136 “Equivocal,” and 73 “Not Consistent with AD.” The [^18^F]FDG-PET showed an AD-like pattern in 73/151 (48.3%) of subjects with CSF “Consistent with AD” and was normal in 30/73 (41.1%) of those with CSF “Not Consistent with AD.” However, 19/151 (12.6%) of individuals with a CSF profile “Consistent with AD” had normal [^18^F]FDG-PET scans (false negatives) whereas 8/73 (11.0%) of those with a CSF profile “Not Consistent with AD” had an AD-like [^18^F]FDG-PET pattern (false positives), resulting in 0.48 sensitivity, 0.84 specificity, and 0.66 AUC of [^18^F]FDG-PET report vs. gold-standard CSF AD biomarkers, and a fair agreement between both tests (κ = 0.334). An AD-like [^18^F]FDG-PET pattern was strongly associated with a CSF “Consistent with AD” (OR = 4.81, *p* < 0.0001) and a lower Amyloid-Tau Index (ATI; β = −0.43, *p* < 0.0001). By region, posterior cingulate gyrus glucose hypometabolism predicted both an AD-like [^18^F]FDG-PET result (OR = 6.41, *p* < 0.0001) and a CSF profile “Consistent with AD” (OR = 2.48, *p* = 0.0003), whereas frontal hypometabolism predicted a Not AD-like [^18^F]FDG-PET result (OR = 5.90, *p* < 0.0001) but also lower odds of a CSF “Not Consistent with AD” (OR = 0.41, *p* = 0.0016).

**Interpretation:**

[^18^F]FDG-PET imaging demonstrated high specificity but limited sensitivity to identify AD as defined by CSF biomarker criteria. Although a report of a typical AD-like [^18^F]FDG-PET pattern of glucose hypometabolism predicted a positive CSF AD biomarker panel, the agreement between [^18^F]FDG-PET report and CSF AD biomarker results was only fair.

**Funding:**

NR-L was supported by a Research Fellowship from the Fundación Ramón Areces, Madrid (Spain). JC, BCD, SEA, PK, and AS-P were supported by the Massachusetts Alzheimer's Disease Research Center (NIH/NIA P30AG062421).

**Research in context**

**Evidence before this study**

We searched PubMed from database inception to December 15th 2025 using combinations of the terms “cerebrospinal fluid,” “Alzheimer” and “positron emission tomography,” restricting inclusion to studies published in English or Spanish. We selected studies that directly compared the diagnostic performance of [^18^F]FDG-PET imaging with CSF biomarkers of Alzheimer's disease (AD), considering the latter as the reference standard. We excluded studies that relied on amyloid PET or blood-based biomarkers for AD diagnosis, as these modalities are not yet widely accessible worldwide. Although several studies reported correlations between [^18^F]FDG-PET-based and CSF biomarker-based diagnoses, all reanalyzed [^18^F]FDG-PET images rather than evaluating its diagnostic performance based on routine narrative reports.

**Added value of this study**

To our knowledge, this is the first study to compare routine narrative reports of [^18^F]FDG-PET brain scans with CSF AD biomarker results in a large memory clinic cohort, thereby assessing the clinical utility of [^18^F]FDG-PET in a real-world tertiary memory clinic setting.

**Implications of all the available evidence**

The observed fair agreement between [^18^F]FDG-PET routine narrative reports and CSF AD biomarker results in this real-world memory clinic setting underscores the need for caution when interpreting [^18^F]FDG-PET reports in isolation. These findings indicate that [^18^F]FDG-PET provides complementary diagnostic information but is not interchangeable with CSF AD biomarkers in the differential diagnosis of age-related cognitive decline.

## Introduction

The diagnosis of Alzheimer's disease (AD) in clinical practice typically relies on a compatible history and neurological examination, including cognitive testing, supported by imaging or fluid biomarkers demonstrating an underlying AD pathophysiological process.^1^, ^2^, ^3^ In 2018, Jack et al. proposed a biomarker-based staging to classify individuals based on their cerebrospinal fluid (CSF) and/or imaging biomarkers of β-amyloidosis, tauopathy, and neurodegeneration—also known as the A/T(N) framework.^4^ Although explicitly developed for research, this framework represented a redefinition of AD from a clinico-biological toward a biological construct. In 2024, based on mounting evidence, Jack et al. updated this framework incorporating blood-based biomarkers and proposing the use of biological definition for AD diagnosis,^5^ a paradigm shift that remains contentious.^6^ While, in the United States, amyloid-β (Aβ) PET imaging has rapidly expanded and replaced CSF AD biomarkers, and blood-based biomarkers are beginning to enter routine clinical practice, CSF AD biomarkers and [^18^F]FDG-PET imaging remain the primary ancillary diagnostic tools available to clinicians worldwide. [^18^F]FDG-PET imaging is considered a biomarker of neurodegeneration that supports the differential diagnosis between AD and related dementias, including the frontotemporal lobar degenerations (FTLDs) and dementia with Lewy bodies (DLB).^7^^,^^8^ Indeed, in the United States, brain [^18^F]FDG-PET imaging is covered by Medicare to discern between AD and FTLD.^9^

Notably, despite the widespread use of [^18^F]FDG-PET imaging in the evaluation of cognitive decline, data on its diagnostic performance against gold-standard CSF AD biomarkers are scarce.^10^, ^11^, ^12^, ^13^ Moreover, in prior studies, one or two nuclear medicine specialists applied visual reads and semi-quantitative methods to re-evaluate retrospective [^18^F]FDG-PET images—conditions likely rendering more uniformity and inflating concordance with CSF AD biomarkers relative to routine clinical practice, where multiple readers with varying experience and reporting styles are usually involved. In this study, we sought to evaluate the real-world agreement between [^18^F]FDG-PET findings and CSF AD biomarkers in a tertiary academic memory clinic and to ascertain the clinician's final diagnosis in extremely discrepant cases (i.e., those with one test normal and the other consistent with AD). To best reflect everyday clinical practice, analyses were based exclusively on the narrative reports of both diagnostic modalities. Our findings underscore the limitations of current reporting practices for [^18^F]FDG-PET brain scans in the context of AD and related dementias.

## Methods

### Study design

This is a chart review-based, cross-sectional, retrospective study in which participants were patients seen at the Mass General Brigham (MGB), an academic medical tertiary referral center in Boston, Massachusetts (USA) with two campuses: Massachusetts General Hospital and Brigham and Women's Hospital, between 2013 and 2025. The study was approved by the Massachusetts General Hospital Institutional Review Board (protocol #2024P002595) and granted exempt research determination for which consent is not required.

### Participants

**Fig. 1:**
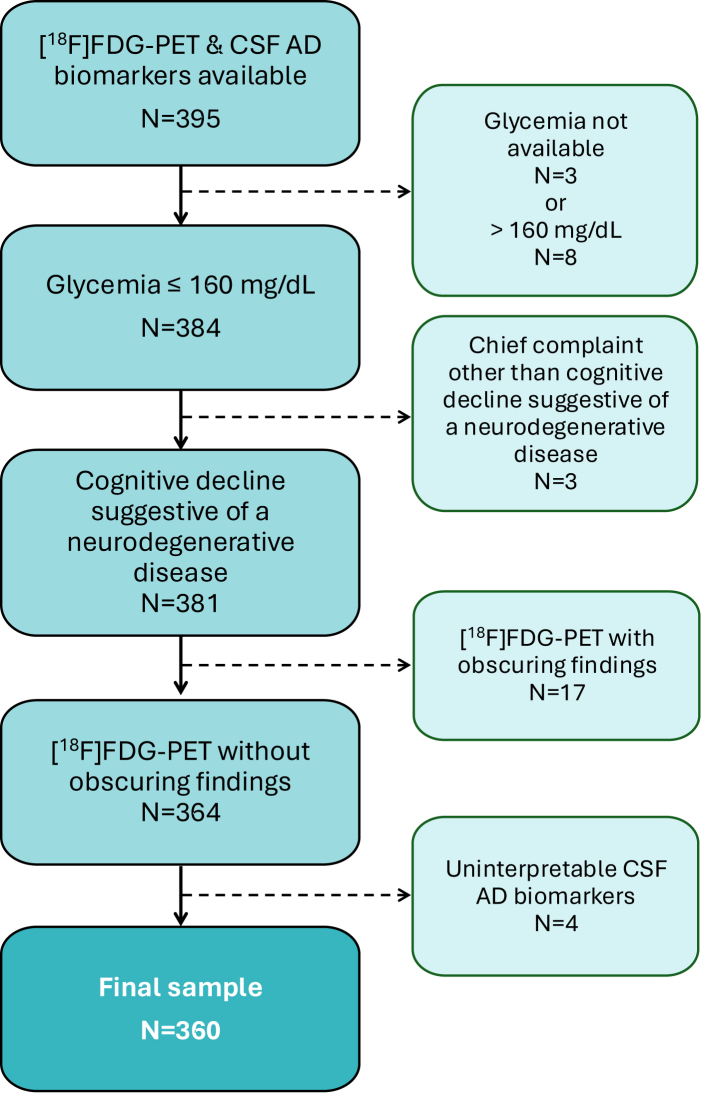
**Flowchart for selection of study participants**.

The MGB electronic health record system was queried to ascertain patients with both [^18^F]FDG-PET brain scan and lumbar puncture with CSF AD biomarker levels between 01/01/2013 and 06/30/2025. Specifically, patients were eligible if they had undergone an [^18^F]FDG-PET brain scan and a lumbar puncture with CSF AD biomarker measurements as part of their diagnostic evaluation of cognitive concerns suggestive of a neurodegenerative disease. Exclusion criteria regarding [^18^F]FDG-PET brain scan were (1) glycemia prior to scan >160 mg/dL^8^^,^^14^ or not available; (2) scan ordered for a chief complaint different from cognitive decline suggestive of a neurodegenerative disease; and (3) abnormal findings obscuring the scan interpretation such as prior stroke, post-traumatic encephalomalacia or post-surgical changes. The only exclusion criterion related to CSF AD biomarkers was uninterpretable results due to values above the upper limit of detection. Flow-chart in Fig. 1 illustrates the study subject selection based on these eligibility criteria.

### Procedures

#### [^18^F]FDG-PET brain scan

[^18^F]FDG-PET brain scans were done following standard procedures.^8^ Patients were asked to fast for at least 5 h and a point-of-care capillary glycemia was obtained prior to the intravenous administration of 2-[^18^F]fluoro-2-deoxy-d-glucose ([^18^F]FDG) (∼6.5 mCi, range 4–15 mCi). Static PET images were acquired 40–90 min post-injection and a non-contrast low-dose helical CT was performed over the same range for attenuation correction and anatomical localization.

#### Data collection

The following data were extracted from MGB electronic health records. Demographic data included age at [^18^F]FDG-PET brain scan, age at lumbar puncture, and sex. Clinical data included MoCA score closest to the [^18^F]FDG-PET scan, clinical presentation (i.e., amnestic vs. non-amnestic, with the latter including executive, behavioral, language, visuospatial, parkinsonism/movement disorders, or other syndromes), and final most likely etiologic diagnosis (see below). MMSE scores closest to the [^18^F]FDG-PET scan were converted into MoCA scores using the conversion table developed by Fasnacht et al.^15^ [^18^F]FDG-PET scan data included date of scan, glycemia immediately prior to the scan, provider ordering the PET scan and department/division, reason for ordering the scan (e.g., suspected neurodegenerative vs. non-neurodegenerative conditions such as epilepsy or autoimmune encephalitis), [^18^F]FDG dose, interval between [^18^F]FDG administration and scan, attending provider signing the report, and narrative results. Four variables were captured from the narrative reports: (1) the diagnosis or diagnoses suggested (if any); (2) the brain regions reported as hypometabolic and their laterality (left or right); and (3) the overall symmetry and laterality of the reported brain hypometabolism (e.g., L > R or R > L). The brain regions annotated were frontal, temporal, parietal, and occipital lobes, insular cortex, anterior cingulate gyrus, posterior cingulate gyrus (PCG), precuneus, basal ganglia, and cerebellum. Whenever a patient had more than one [^18^F]FDG-PET brain scan available, only the closest to the lumbar puncture was considered.

CSF data extracted included the CSF AD biomarker assay used—either the Athena ADmark™ panel [Athena Diagnostics, Worcester, MA] or the Roche Elecsys™ panel [Mayo Clinic Department of Laboratory Medicine and Pathology, Rochester, MN]; the date of the lumbar puncture; the final interpretation noted in the report (see below); and the CSF AD biomarker levels (i.e., Aβ_42_, p-tau181, and total tau levels as well as the “Aβ_42_ to total tau index” or ATI—calculated as Aβ_42_/[240 + 1.18 × total tau]—in the Athena ADmark™ panel or Aβ_42_, p-tau181, and total tau levels as well as the Aβ_42_/p-tau181 ratio in the Roche Elecsys™ panel).

### Outcomes

#### CSF biomarker-based determination of AD diagnosis

The diagnosis of AD was based on the CSF AD biomarker panel results, which are gold-standard FDA-approved diagnostic tests. Thus, a patient was considered to have definite AD if the CSF AD biomarker panel was “Consistent with AD” with either the Athena ADmark™ or the Roche Diagnostics Elecsys™ assays. For the Athena ADmark™ assay, a result is reported as “Consistent with AD” if the ATI [Aβ_42_/(240 + 1.18 × total tau)] is <0.8 and p-tau181 is >68 pg/mL; “Not Consistent with AD” if the ATI is >1.2 and p-tau181 is <54 pg/mL; “Borderline” if the ATI is 0.8–1.2 and/or p-tau181 is 54–68 pg/mL; and “Indeterminate” if the results do not fit in any of the other three categories. For this study, we combined the “Borderline” and “Indeterminate” results in an “Equivocal” category. Following our eligibility criteria, two participants were excluded because their total tau levels with the Athena ADmark™ assay were 3512.4 and 4547.6 pg/mL.

The Roche Diagnostics Elecsys™ assay result is “Consistent with AD” when the p-tau181/Aβ_42_ ratio is >0.023 (or >0.028 for results after May 31, 2023) and “Not Consistent with AD” if the p-tau181/Aβ_42_ ratio is ≤0.023 (or ≤0.028 for results after May 31, 2023). The normal AD biomarker levels for this assay are: Aβ_42_ >1026 pg/mL (>834 pg/mL after May 31, 2023), p-tau181 ≤21.7 pg/mL (≤21.6 pg/mL after May 31, 2023), and total tau ≤238 pg/mL. For the purpose of this study, individuals with low Aβ_42_ but normal p-tau181 (resulting in normal p-tau181/Aβ_42_ ratio, n = 7); those with low Aβ_42_, normal p-tau181 but elevated p-tau181/Aβ_42_ ratio (n = 9); and those with p-tau181 or Aβ_42_ below the lower level of detection (<8 pg/mL or <250 pg/mL, respectively, n = 4) were assigned to an “Equivocal” category. In subjects with p-tau181 or Aβ_42_ below the lower level of detection, the p-tau181/Aβ_42_ ratio was calculated based on those lower limits of detection. Following our eligibility criteria, two participants were excluded because their Aβ_42_ level with the Roche Elecsys™ assay was above the upper limit of detection (>1700 pg/mL) so their p-tau181/Aβ_42_ ratio could not be calculated.

#### [^18^F]FDG-PET brain scan-based determination of AD diagnosis

A total of 31 participants were excluded on the basis of their [^18^F]FDG-PET eligibility criteria: three due to glycemia unavailable, eight to hyperglycemia > 160 mg/dL, three to chief complaint other than cognitive decline suggestive of a neurodegenerative disease (two for seizure work-up and one for suspected anti-LGI1 limbic encephalitis), and 17 due to abnormal findings potentially obscuring the interpretation (four due to encephalomalacia of unknown cause; four to stroke; two to each of normal pressure hydrocephalus, post-surgical changes, limbic encephalitis and medication side effect; and one to a prior subdural hematoma). We categorized the [^18^F]FDG-PET results of the remaining participants as follows. If the narrative impression concluded normal metabolism or normal for age and atrophy, we adjudicated a “Normal” [^18^F]FDG-PET scan. The remaining patients were considered to have an “Abnormal” [^18^F]FDG-PET scan. For patients in whom a single final diagnosis was recorded in the summary impression of the [^18^F]FDG-PET scan report, this was categorized as “AD-like” or “Not AD-like.” Atypical AD patterns corresponding to atypical AD clinical syndromes such as logopenic variant primary progressive aphasia (lvPPA) and posterior cortical atrophy (PCA) were included in the AD-like category if they were reported as a single diagnosis, whereas non-AD patterns (i.e., FTLD-like, DLB-like) were separated in an abnormal “Not AD-like” category. [^18^F]FDG-PET brain scans that had more than one possible diagnosis in the summary impression, or were reported abnormal but lacked any specific diagnosis, or had too widespread and severe hypometabolism to render a diagnosis were categorized as abnormal “Inconclusive.” We investigated the interrater reliability between two neurologists (NR-L and AS-P) for the AD-like vs. other (combining Normal, Not AD-like, and Inconclusive) result categories in 100 randomly selected [^18^F]FDG-PET reports and estimated a kappa of 0.863 (95% CI [0.757–0.968]), consistent with an almost perfect agreement.

### Mapping of regional hypometabolism by [^18^F]FDG-PET scan or CSF AD biomarker result groups

Hypometabolic areas reported across [^18^F]FDG-PET or CSF AD biomarker result groups were represented in axial, sagittal, and coronal brain maps using the MINC Toolkit (https://github.com/BIC-MNI/minc-toolkit-v2), with the color scale illustrating the proportion (%) of individuals within that [^18^F]FDG-PET or CSF AD biomarker result category who were reported to have hypometabolism in each specific brain area. The regions annotated included left and right frontal, temporal, parietal, and occipital lobes, insular cortex, anterior cingulate gyrus, PCG, precuneus, and basal ganglia (Table S2).

### Association of clinical presentation with final etiologic diagnosis through [^18^F]FDG-PET scan or CSF AD biomarker results

To visualize the association between clinical syndromic presentation, [^18^F]FDG-PET scan results, CSF AD biomarker results, and final etiologic diagnosis, we generated Sankey diagrams using SankeyMATIC (https://sankeymatic.com) with these variables as nodes and the flows between the nodes colored following a “each flow's target” direction. To simplify this diagram, the clinical syndromic presentation was classified as amnestic vs. non-amnestic. The final etiologic diagnoses were categorized as AD, AD-plus (encompassing differential diagnoses between AD and another neurodegenerative or non-neurodegenerative cause as well as mixed AD and another neurodegenerative or non-neurodegenerative copathology), FTLD, DLB, other neurodegenerative diagnoses (with or without another alternative or concurrent neurodegenerative or non-neurodegenerative diagnosis), and other non-neurodegenerative diagnoses. We also generated another Sankey diagram to illustrate the association of each non-amnestic syndromic presentation and the final etiologic diagnosis, without the [^18^F]FDG-PET scan and CSF AD biomarker results.

### Statistical analysis

The statistical analyses were conducted in GraphPad Prism version 10.1 (Dotmatics, Boston, MA) and STATA version 15.0 (STATACorp, LLC, College Station, TX). Graphs were performed with GraphPad Prism version 10.1 (Dotmatics, Boston, MA). As described above, for the purpose of statistical analyses, CSF results were categorized as Not Consistent with AD, Consistent with AD, or Equivocal (i.e., Athena ADmark™ Borderline and Indeterminate results were grouped as Equivocal), whereas [^18^F]FDG-PET results were categorized as Normal or Abnormal and these were further subdivided into AD-like, Not AD-like, and Inconclusive. To describe the cohort, continuous variables (e.g., age, CSF AD biomarker levels) were compared across CSF- and [^18^F]FDG-PET-based diagnostic groups with one-way or Kruskal–Wallis ANOVA followed by Tukey's or Dunn's post-test for normally and non-normally distributed data, respectively. Categorical variables (e.g., sex) were compared across CSF- and [^18^F]FDG-PET-based diagnostic groups with the Fisher's exact test.

To evaluate the performance (sensitivity, specificity, positive and negative predictive values, likelihood ratio, and area under the curve [AUC]) of [^18^F]FDG-PET against the gold standard CSF AD biomarker results, we built a 2 × 2 contingency table with CSF Consistent with AD vs. other CSF groups (Not Consistent with AD and Equivocal) and AD-like vs. other [^18^F]FDG-PET groups (combining Normal, Not AD-like, and Inconclusive patterns). We also examined the agreement between both diagnostic tests using the kappa agreement test. To investigate a possible information bias in the interpretation of the [^18^F]FDG-PET brain scan due to availability of CSF AD biomarker results, we divided the whole sample in subjects who underwent the [^18^F]FDG-PET brain scan before the lumbar puncture and those who did it after the lumbar puncture and compared sensitivity, specificity, predictive values, AUC, and kappa agreement between both subsamples. For these sensitivity analyses, n = 14 subjects who underwent both procedures on the same day were analyzed as if the [^18^F]FDG-PET brain scan was performed before the lumbar puncture.

To further examine the correlation between [^18^F]FDG-PET brain scan and CSF AD biomarker results, we built a logistic regression model with CSF AD biomarker results as dependent variable (combining Not Consistent with AD and Equivocal as reference), [^18^F]FDG-PET results as independent variable (combining Normal, Not-AD-like, and Inconclusive patterns as reference), and controlling for age, sex, and the time interval between [^18^F]FDG-PET brain scan and lumbar puncture. Similarly, we performed a multivariable linear regression with CSF ATI as dependent variable, [^18^F]FDG-PET results as independent variable (combining Normal, Not AD-like, and Inconclusive patterns as reference) and age, sex, and the interval between [^18^F]FDG-PET scan and lumbar puncture as co-variates. Lastly, we applied logistic regression models to assess whether specific hypometabolic brain areas predicted an AD-like [^18^F]FDG-PET and a CSF Consistent with AD or, instead, a Not AD-like [^18^F]FDG-PET and a CSF Not Consistent with AD. In these analyses, the [^18^F]FDG-PET or CSF AD biomarker results were treated as dependent variable and PCG or frontal hypometabolism (any, i.e., left, right or both frontal lobes) as independent variable, controlling for age, sex, and the time interval between [^18^F]FDG-PET brain scan and lumbar puncture.

### Role of the funding source

The funding sources of the study had no role in study design, data collection, data analysis, data interpretation, or writing of the report.

## Results

### Characteristics of study participants

The flow-chart in Fig. 1 illustrates study subject selection based on our pre-specified eligibility criteria. A total of 360 patients were included in downstream analyses. Notably, [^18^F]FDG-PET brain scan reports were ordered by 139 different clinicians and signed by 28 different specialist readers between 2013 and 2025. Of the 360 reports, 264 were signed by a nuclear medicine attending physician and 96 by a neuroradiology attending physician, 237 were reviewed by two attending physicians (typically a neuroradiologist and a nuclear medicine specialist), and 184 were drafted by a trainee. Of the 360 patients, 311 (86.4%) had the Athena ADmark™ CSF biomarker panel, whereas 49 (13.6%) had the Roche Diagnostics Elecsys™ CSF biomarker panel.

**Fig. 2:**
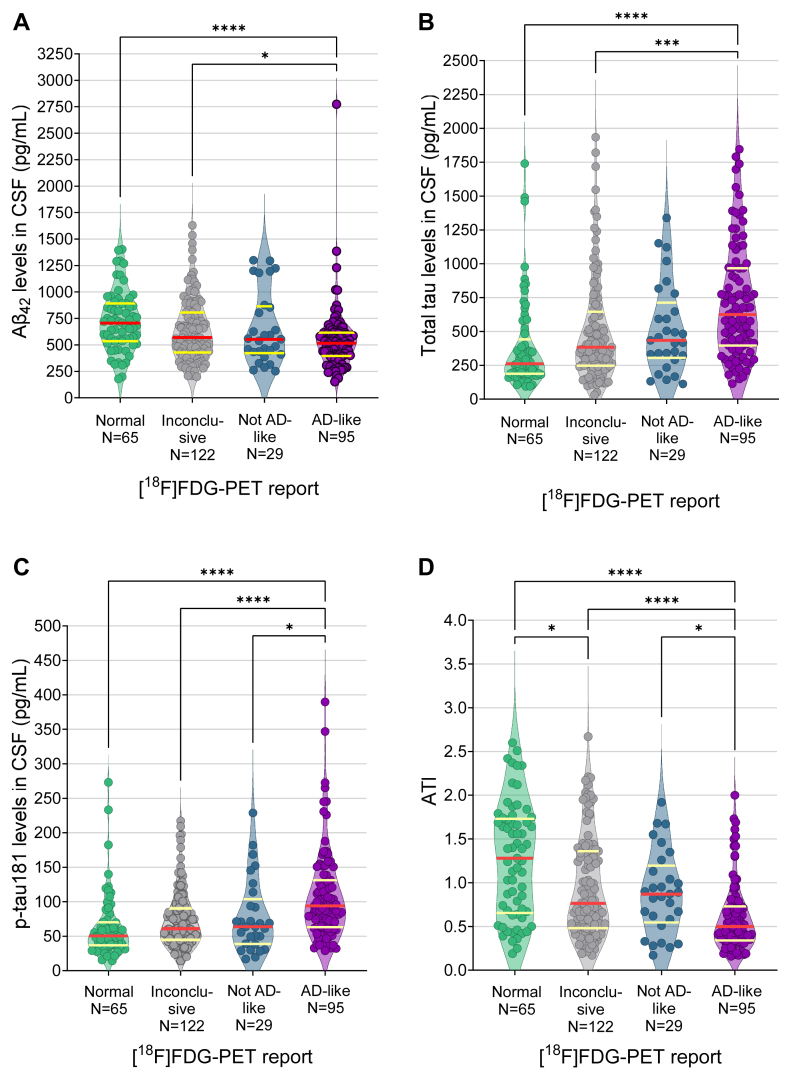
**CSF AD biomarker levels across [^18^F]FDG-PET report groups.** Comparison of Aβ_42_ (**A**), total tau (**B**), p-tau181 (**C**), and ATI (**D**) across [^18^F]FDG-PET result groups. Only subjects with Athena ADmark CSF panel are represented. Red and yellow lines indicate median and interquartile ranges, respectively. ∗*p* < 0.05; ∗∗∗*p* < 0.001; ∗∗∗∗*p* < 0.0001; Kruskal–Wallis ANOVA followed by Dunn's post-test. Abbreviations: [^18^F]FDG = 2-[^18^F]fluoro-2-deoxy-d-glucose; Aβ = Amyloid-beta; AD = Alzheimer's disease; ATI = Amyloid-Tau Index; CSF = cerebrospinal fluid; p-tau = phospho-tau; PET = Positron Emission Tomography.

Tables 1 and 2 depict the characteristics of study participants split by CSF AD biomarker and [^18^F]FDG-PET scan results, respectively. Based on CSF AD biomarker profiles (gold-standard), 73 (20.2%) patients were classified as Not Consistent with AD, 136 (37.8%) as Equivocal, and 151 (41.9%) as Consistent with AD (Table 1). Based on [^18^F]FDG-PET scan results, 75 (20.8%) were classified as Normal, 107 (29.7%) as Abnormal AD-like, 37 (10.3%) as Abnormal Not AD-like, and 141 (39.2%) as Abnormal Inconclusive (Table 2). Among the 107 subjects with an AD-like [^18^F]FDG-PET, 11 had a [^18^F]FDG-PET compatible with atypical AD (six lvPPA, four PCA, and one atypical AD non-otherwise specified). Regarding the 37 with an Abnormal Not AD-like [^18^F]FDG-PET pattern, 31 were reported as suggestive of FTLD, five as DLB, and one as Parkinson's disease. Among the 141 subjects with an Abnormal Inconclusive [^18^F]FDG-PET pattern, 63 had more than one diagnosis, 70 had no specific diagnosis, and nine had too extensive hypometabolism to render a diagnosis. The comparisons of CSF AD biomarker levels across [^18^F]FDG-PET groups showed significantly lower Aβ_42_ levels and ATI in AD-like vs. Normal and Inconclusive [^18^F]FDG-PET groups, significantly higher levels of p-tau181 in the AD-like vs. the Normal, Inconclusive, and Not AD-like [^18^F]FDG-PET groups, and significantly higher levels of total tau in the AD-like vs. the Normal and Inconclusive [^18^F]FDG-PET groups (Table 1 and Fig. 2).

**Table 1:** Characteristics of study participants by CSF AD biomarker results.

	Total	Not consistent with AD	Equivocal	Consistent with AD	*P*-value
**N**	360	73	136	151	N.A.
**Sex**, n (%) female	150 (41.7)	26 (35.6)	53 (39.0)	71 (47.0)	0.1904
**Age at [^18^F]FDG-PET** (y)	66.8 ± 9.1	65.7 ± 9.8	67.9 ± 8.7	66.3 ± 9.0	0.1547
**Age at LP** (y)	66.9 ± 9.0	65.8 ± 9.8	67.9 ± 8.5	65.7 ± 9.4	0.1712
**[^18^F]FDG-PET vs. LP interval** (mo)	2.0 (0.0–6.0)	2.0 (1.0–6.0)	2.0 (0.0–8.0)	1.0 (0.0–4.0)	**0.0232**
**Clinical presentation**, n (%):					
***Amnestic***	203 (56.4)	34 (46.6)	72 (52.9)	97 (64.2)	**0.0252**
***Non-amnestic***	157 (43.6)	39 (53.4)	64 (47.1)	54 (35.8)	
**Baseline MoCA score**	19.9 ± 5.9	21.9 ± 5.2	19.78 ± 6.5	18.91 ± 5.5	**0.0005**
**Interval baseline MoCA–[^18^F]FDG-PET** (mo)	1.0 (0.0–4.3)	2 (0.0–5.0)	2 (0.0–5.5)	1 (0.0–3.0)	0.3322
**Interval baseline MoCA–LP** (mo)	3 (1.0–8.0)	3 (1.0–10.0)	3 (1.0–8.0)	3 (1.0–6.8)	0.7731
**Glycemia** (mg/dL)	101.2 ± 14.9	101.5 ± 17.2	102.9 ± 14.8	100.2 ± 13.7	0.1005
**[^18^F]FDG-PET dose** (mCi)	6.5 ± 2.3	7.1 ± 2.6	6.5 ± 2.3	6.2 ± 2.1	0.1015
**Scan time after [^18^F]FDG dose** (min)	46.3 ± 5.5	47.4 ± 7.6	46.0 ± 4.2	46.1 ± 5.8	0.2897
**Aβ_42_** (pg/mL)	633.1 ± 305.0	814.1 ± 196.1	681.3 ± 405.4	503.0 ± 147.6	**<0.0001**
**total tau** (pg/mL)	545.3 ± 397.4	209.7 ± 196.1	364.2 ± 405.4	868.4 ± 147.6	**<0.0001**
**p-tau181** (pg/mL)	83.2 ± 55.3	37.9 ± 10.5	59.0 ± 23.2	126.7 ± 57.7	**<0.0001**
**ATI**	0.9 ± 0.6	1.7 ± 0.4	1.0 ± 0.5	0.4 ± 0.2	**<0.0001**
**p-tau181/Aβ_42_ ratio**	0.041 ± 0.032	0.012 ± 0.003	0.028 ± 0.012	0.068 ± 0.032	**<0.0001**

Categorical variables are expressed as n (%) and compared using Fisher's exact test. Continuous variables are expressed as mean ± SD (except time intervals, which are expressed in median (interquartile range) using absolute values) and analyzed using Kruskal–Wallis ANOVA followed by Dunn's post-test. Statistically significant results are bold-faced. MoCA score was not available for n = 62 individuals (n = 12 with CSF Not Consistent with AD, n = 23 Equivocal, and n = 27 Consistent with AD). The ATI is calculated as Aβ_42_/(240 + 1.18 × total tau).

Abbreviations: [^18^F]FDG = 2-[^18^F]fluoro-2-deoxy-d-glucose; Aβ = amyloid-beta; AD = Alzheimer's disease; ATI = Amyloid-Tau Index; CSF = cerebrospinal fluid; LP = lumbar puncture; MoCA = Montreal cognitive assessment; N.A. = not applicable; p-tau = phospho-tau; PET = Positron Emission Tomography.

Note: Aβ_42_, total tau, p-tau181, and ATI results correspond to n = 311 individuals with the Athena ADmark™ CSF panel (n = 63 for Not AD, n = 117 for Equivocal, and n = 131 for Consistent with AD), whereas the p-tau181/Aβ_42_ ratio corresponds to n = 49 patients with the Roche Elecsys™ panel (n = 10 for Not AD, n = 19 for Equivocal, and n = 20 for Consistent with AD).

**Table 2:** Characteristics of study participants split by [^18^F]FDG-PET results.

	Total	Normal	Abnormal			*P*-value
			Inconclusive	Not AD-like	AD-like	
**N**	360	75	141	37	107	N.A.
**Sex**, n (%) female	150 (41.7)	31 (41.3)	56 (39.7)	20 (54.1)	43 (40.2)	0.4526
**Age at [^18^F]FDG-PET** (y)	66.8 ± 9.1	68.2 ± 9.4	67.1 ± 9.5	66.9 ± 7.8	65.2 ± 8.4	0.1259
**Age at LP** (y)	66.9 ± 9.0	68.3 ± 9.4	67.2 ± 9.4	67.0 ± 7.6	65.5 ± 8.5	0.1739
**[^18^F]FDG-PET vs. LP interval** (mo)	2.0 (0.0–6.0)	2.0 (0.0–8.0)	1.0 (0.0–4.0)	2.0 (0.0–6.0)	2.0 (0.0–6.0)	0.5164
**Clinical presentation**, n (%):						
***Amnestic***	203 (56.4)	43 (57.3)	82 (58.2)	14 (37.8)	64 (59.8)	0.1224
***Non-amnestic***	157 (43.6)	32 (42.7)	59 (41.8)	23 (62.0)	43 (40.2)	
**Baseline MoCA score**	19.9 ± 5.9	22.6 ± 5.2	20.0 ± 5.6	20.6 ± 4.6	17.8 ± 6.4	**<0.0001**
**Interval baseline MoCA–[^18^F]FDG-PET** (mo)	1.0 (0.0–4.3)	2.0 (0.0–8.0)	2 (0.0–5.0)	1 (0.0–4.0)	1 (0.0–3.0)	0.1790
**Interval baseline MoCA–LP** (mo)	3 (1.0–8.0)	2.5 (0.8–6.0)	3.0 (1.0–10.3)	3.0 (1.0–8.0)	3.0 (1.0–7.0)	0.6254
**Glycemia** (mg/dL)	101.2 ± 14.9	99.2 ± 15.3	102.6 ± 15.9	103.1 ± 14.6	101.0 ± 13.1	0.2359
**[^18^F]FDG dose** (mCi)	6.5 ± 2.3	6.7 ± 2.4	6.389 ± 2.2	7.02 ± 2.7	6.3 ± 2.3	0.2684
**Scan time after [^18^F]FDG dose**	46.3 ± 5.5	46.0 ± 4.7	46.6 ± 5.8	48.1 ± 9.5	45.6 ± 3.5	0.1768
**Laterality**, n (%) bilateral	251 (88)	N.A.	127 (91)	30 (81)	94 (88)	0.2636
*Bilateral symmetric, n (%)*	100 (35)	N.A.	63 (45)	11 (30)	26 (24)	
*Bilateral asymmetric R > L, n (%)*	66 (23)	N.A.	26 (18)	8 (22)	32 (30)	
*Bilateral asymmetric L > R, n (%)*	85 (30)	N.A.	38 (27)	11 (30)	36 (34)	
*Unilateral R, n (%)*	9 (3)	N.A.	6 (4)	0 (0)	2 (2)	
*Unilateral L, n (%)*	24 (9)	N.A.	7 (5)	7 (19)	11 (10)	

Categorical variables are expressed as n (%) and analyzed using Fisher's exact test. Continuous variables are expressed as mean ± SD (except time intervals, which are expressed as median (interquartile range) in absolute values) and analyzed using Kruskal–Wallis ANOVA with Dunn's post-test. Statistically significant results are bold-faced. MoCA score was not available for n = 62 individuals (n = 17 with [^18^F]FDG-PET scan Normal, n = 23 Inconclusive, n = 10 Not AD-like, and n = 12 AD-like).

Abbreviations: [^18^F]FDG = 2-[^18^F]fluoro-2-deoxy-d-glucose; AD = Alzheimer's disease; L = left; LP = lumbar puncture; MoCA = Montreal cognitive assessment; N.A. = not applicable; PET = Positron Emission Tomography; R = right.

Note: Laterality sample sizes and % do not include individuals with normal [^18^F]FDG-PET scan.

### Agreement between [^18^F]FDG-PET brain scan and CSF AD biomarker results

**Fig. 3:**
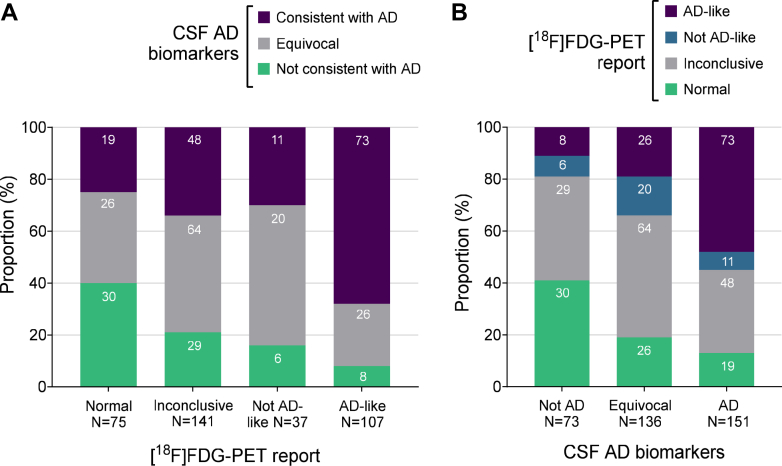
**Agreement between brain [^18^F]FDG-PET scan reports and CSF AD biomarker results.** Stacked bar plots show (**A**) the proportion of CSF AD biomarker categories across [^18^F]FDG-PET report interpretations, and (**B**) the proportion of [^18^F]FDG-PET report results across CSF AD biomarker categories. Absolute numbers of individuals in each category are depicted in white font within the bars. Abbreviations: [^18^F]FDG = 2-[^18^F]fluoro-2-deoxy-d-glucose; AD = Alzheimer's disease; PET = Positron Emission Tomography.

Notably, a Normal [^18^F]FDG-PET result was more frequent in the CSF Not Consistent with AD group (30/73, 41.1%), whereas the AD-like [^18^F]FDG-PET result was the predominant pattern in the CSF Consistent with AD group (73/151, 48.3%) (Fig. 3). However, surprisingly, 19 of 151 (12.6%) individuals with a CSF Consistent with AD had a Normal [^18^F]FDG-PET scan (false negatives), and 8 of 73 (11.0%) with a CSF Not Consistent with AD had an AD-like [^18^F]FDG-PET pattern (false positives) (Fig. 3). Also, of note, the CSF Equivocal group contained a mix of Normal and AD-like [^18^F]FDG-PET results (26/136, 19.1% for each category), and the proportions of Not AD-like and Inconclusive [^18^F]FDG-PET patterns were relatively constant across all three CSF AD biomarker groups (Fig. 3).

When we binarized [^18^F]FDG-PET brain scan results as AD-like vs. other patterns and CSF AD biomarker results as Consistent with AD vs. other, the [^18^F]FDG-PET brain scan demonstrated a sensitivity of 0.48 (95% CI: 0.41–0.56) and a specificity of 0.84 (95% CI: 0.78–0.88) for identifying CSF-defined AD. The agreement between [^18^F]FDG-PET-based and CSF-based binary classifications was only fair (69% agreement, κ coefficient [95% CI] = 0.334 [0.237–0.431]) (Table 3). Positive and negative likelihood ratios were relatively small (LR+ 2.972 and LR− 0.617, respectively), indicating that an AD-like [^18^F]FDG-PET pattern only increases ∼3 times the probability of having CSF biomarker results Consistent with AD and that an [^18^F]FDG-PET pattern different from AD-like only reduces ∼40% the probability of having CSF biomarker results Consistent with AD (Table 3). As sensitivity analysis, we compared the performance of [^18^F]FDG-PET brain scan against the Athena ADmark™ panel (n = 311) vs. the Roche Elecsys™ assay separately; while results were largely comparable, the [^18^F]FDG-PET brain scan appeared to have a slightly better performance when using the Roche Elecsys™ CSF biomarker panel as gold-standard (Table S1).

**Table 3:** Contingency tables, sensitivity, specificity, and positive and negative predictive values of [^18^F]FDG-PET relative to CSF AD biomarker results.

All (n = 360)					
		**[^18^F]FDG-PET**			Sensitivity: 0.48 (0.41–0.56) Specificity: 0.84 (0.78–0.88) PPV: 0.68 (0.59–0.76) NPV: 0.69 (0.63–0.75) LR+: 2.972; LR−: 0.617 AUC: 0.660 (0.610–0.710) Agreement: 68.9% Kappa: 0.334 (0.237–0.431)
		**AD-like**	**Other**		
**CSF**	**AD**	73 (68.2)	78 (30.8)	151	
	**Other**	34 (31.8)	175 (69.2)	209	
		107	253	360	
**[^18^F]FDG-PET before LP (n** = **232)**					

		**[^18^F]FDG-PET**			Sensitivity: 0.45 (0.37–0.54) Specificity: 0.81 (0.72–0.87) PPV: 0.73 (0.62–0.81) NPV: 0.56 (0.48–0.64) LR+: 2.323; LR−: 0.681 AUC: 0.629 (0.571–0.686) Agreement: 61.6% Kappa: 0.250 (0.137–0.363)
		**AD-like**	**Other**		
**CSF**	**AD**	56 (72.7)	68 (43.9)	124	
	**Other**	21 (27.3)	87 (56.1)	108	
		77	155	232	

**[^18^F]FDG-PET after LP (n** = **128)**					
		**[^18^F]FDG-PET**			Sensitivity: 0.63 (0.44–0.78) Specificity: 0.87 (0.79–0.92) PPV: 0.57 (0.39–0.73) NPV: 0.90 (0.82–0.94) LR+: 4.892; LR−: 0.425 AUC: 0.751 (0.652–0.849) Agreement: 82.0% Kappa: 0.481 (0.300–0.663)
		**AD-like**	**Other**		
**CSF**	**AD**	17 (56.7)	10 (10.2)	27	
	**Other**	13 (43.3)	88 (89.8)	101	
		30	98	128	

Ranges in parentheses represent 95% confidence intervals. [^18^F]FDG-PET before LP means PET scan performed before the LP or on the same day. [^18^F]FDG-PET after LP means PET scan performed the day after the LP or later. Abbreviations: AD = Alzheimer's disease; AUC = area under the receiver operating characteristic curve; CSF = cerebrospinal fluid; [^18^F]FDG = 2-[^18^F]fluoro-2-deoxy-d-glucose; LP = lumbar puncture; LR = likelihood ratio; NPV = negative predictive value; PET = Positron Emission Tomography; PPV = positive predictive value.

We hypothesized that the availability of gold-standard CSF AD biomarker results prior to the [^18^F]FDG-PET scan may have influenced the reporting of the latter and, thus, the concordance between both tests. To examine the effect of the interval between [^18^F]FDG-PET scan and lumbar puncture on the agreement between the two tests, we split the total sample (n = 360) in two subgroups: those with [^18^F]FDG-PET scan performed prior to or the same day as the lumbar puncture (n = 232) and those with their [^18^F]FDG-PET scan performed after their lumbar puncture (n = 128), and run similar analyses. Indeed, the diagnostic accuracy of [^18^F]FDG-PET scan was considerably higher when it was performed after the lumbar puncture compared to when it was done prior or on the same day. Specifically, the sensitivity improved from 0.45 to 0.63, the specificity from 0.81 to 0.87, the agreement from 62% to 82%, the LR+ from 2.323 to 4.892, the LR− from 0.681 to 0.425, the AUC from 0.629 to 0.751, and the κ coefficient from 0.250 (fair agreement) to. 0.481 (moderate agreement) (Table 3).

### Associations between [^18^F]FDG-PET brain scan and CSF AD biomarker results

We next investigated the association between [^18^F]FDG-PET scan and CSF AD biomarker results via logistic and multivariable linear regression analyses. When controlling for age at [^18^F]FDG-PET scan, sex, and time interval between [^18^F]FDG-PET scan and lumbar puncture, an AD-like [^18^F]FDG-PET pattern was significantly associated with a CSF Consistent with AD (OR [95% CI] = 4.81 [2.95–7.99], *p* < 0.0001). Similarly, an AD-like [^18^F]FDG-PET pattern was significantly associated with a lower CSF ATI (β = −0.43, *p* < 0.0001), holding constant age at [^18^F]FDG-PET scan, sex, and interval between both tests (Table 4).

**Table 4:** Associations between [^18^F]FDG-PET scan results and CSF AD biomarker results or CSF ATI.

	CSF AD biomarker results *(Consistent with AD vs. Not Consistent with AD/Equivocal)*			CSF ATI		
	OR	95% CI	*P*-value	β	95% CI	*P*-value
**[^18^F]FDG-PET results** *(AD-like vs. Normal/Inconclusive/Not AD-like)*	**4.81**	**2.95–7.99**	**<0.0001**	**−0.43**	**−0.56 to −0.30**	**<0.0001**
**Age** (y)	1.00	0.98–1.03	0.9911	**−0.01**	**−0.01–0.00**	**0.0214**
**Sex** (*Female as ref.*)	0.64	0.40–1.01	0.0545	**0.15**	**0.02–0.27**	**0.0196**
**[^18^F]FDG-PET vs. LP interval** (mo)	1.02	1.00–1.05	0.0616	−0.004	−0.009–0.002	0.2215

Statistically significant results are bold-faced.

The ATI is calculated as Aβ_42_/(240 + 1.18 × total tau). [^18^F]FDG-PET vs. LP interval was treated as an interval variable, that is, as a continuous variable with negative values when the LP preceded the [^18^F]FDG-PET brain scan.

Abbreviations: AD = Alzheimer's disease; ATI = Amyloid-Tau Index; CI = confidence interval; CSF = cerebrospinal fluid; [^18^F]FDG = 2-[^18^F]fluoro-2-deoxy-d-glucose; LP = lumbar puncture; OR = odds ratio; PET = Positron Emission Tomography.

**Fig. 4:**
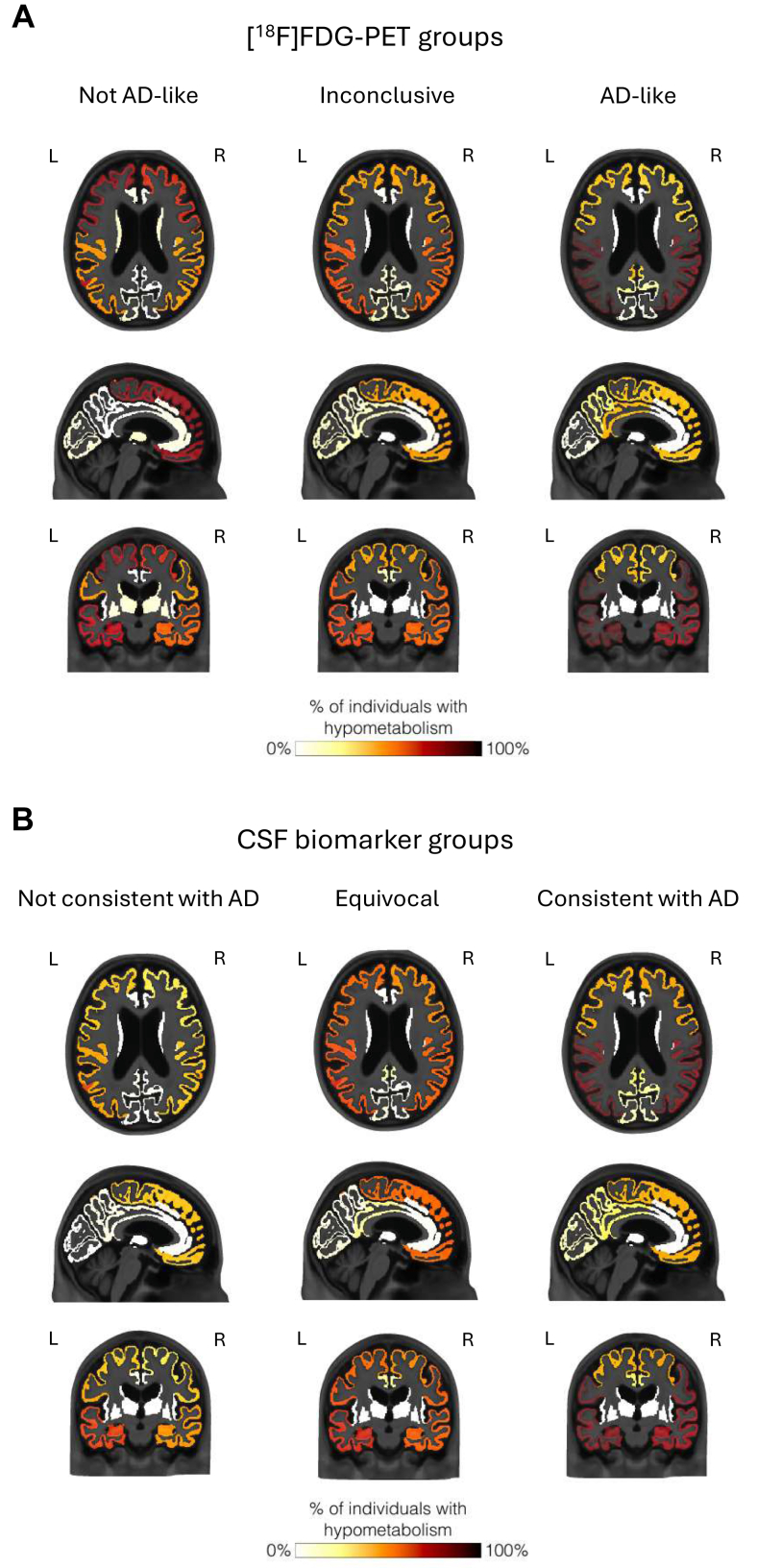
**Relationship between reported regional hypometabolism patterns and patient groups as classified by [^18^F]FDG-PET and CSF AD biomarker results.** Narrative reports of regional hypometabolism were used to generate maps of the proportions of individuals with hypometabolism in each brain region in each group. Patients were grouped as (**A**) “AD-like,” “Not AD-like,” or “Inconclusive” based on their [^18^F]FDG-PET narrative reports, and as (**B**) “Consistent with AD,” “Equivocal,” or “Not Consistent with AD” based on their CSF AD biomarker panel result. Note: subjects with Normal [^18^F]FDG-PET were excluded from this analysis. Abbreviations: AD = Alzheimer's disease; CSF = cerebrospinal fluid; FDG = 2-[^18^F]fluoro-2-deoxy-D-glucose; L = left; PET = Positron Emission Tomography; R = right.

To visualize how the regional pattern of hypometabolism influences the report of an AD-like vs. other [^18^F]FDG-PET patterns, we mapped the proportion of subjects with reported hypometabolism in each brain region onto coronal, axial and sagittal brain templates and compared these maps across [^18^F]FDG-PET report patterns. As expected, the AD-like [^18^F]FDG-PET result was characterized by very frequent report of symmetric temporo-parietal hypometabolism, followed by PCG and precuneus hypometabolism. In comparison, the Not AD-like [^18^F]FDG-PET pattern had more frequent frontal involvement and more asymmetry between hemispheres, and the Inconclusive pattern resembled a mix between AD-like and Not AD-like patterns (Fig. 4A). To further examine whether these apparent regional differences significantly impact the final [^18^F]FDG-PET interpretation, we compared the strength of the association between PCG vs. frontal lobe hypometabolism and an AD-like or a Not AD-like [^18^F]FDG-PET result. We selected these regions because PCG is known to be an AD-vulnerable area,^16^^,^^17^ whereas frontal lobes are affected earlier in FTLD.^18^ We found a strong and statistically significant association between PCG hypometabolism and an AD-like [^18^F]FDG-PET result; specifically, individuals with hypometabolism in the PCG were more than six times more likely to receive an AD-like [^18^F]FDG-PET result and ∼85% less likely to receive a Not AD-like result than individuals with preserved or non-reported PCG metabolism (Table 5 and Fig. 4A). In sharp contrast with the PCG, the presence of frontal hypometabolism was not significantly associated with an AD-like [^18^F]FDG-PET result; instead, individuals with reported frontal hypometabolism were six times more likely to receive a Not AD-like [^18^F]FDG-PET result than those with preserved or non-reported frontal metabolism (Table 5 and Fig. 4A).

**Table 5:** Associations between hypometabolism in posterior cingulate gyrus or frontal lobes and [^18^F]FDG-PET or CSF AD biomarker results.

	[^18^F]FDG-PET results					
	AD-like vs. Not AD-like/Inconcl./Normal			Not AD-like vs. AD-like/Inconcl./Normal		
	OR	95% CI	*P*-value	OR	95% CI	*P*-value
PCG hypometabolism	**6.41**	**3.81–10.96**	**<0.0001**	**0.16**	**0.02–0.53**	**0.0011**
Age (y)	0.98	0.95–1.01	0.1031	1.00	0.96–1.04	0.9422
Sex (Female as ref.)	1.17	0.71–1.95	0.5494	0.56	0.28–1.13	0.1055
[^18^F]FDG-PET vs. LP interval (mo)	**1.03**	**1.00–1.05**	**0.0368**	0.99	0.96–1.02	0.4166
	**OR**	**95% CI**	***P*-value**	**OR**	**95% CI**	***P*-value**

Frontal hypometabolism	1.26	0.79–2.02	0.3279	**5.90**	**2.62–15.12**	**<0.0001**
Age (y)	0.98	0.95–1.00	0.0536	1.01	0.97–1.05	0.6650
Sex (Female as ref.)	1.19	0.74–1.93	0.4696	0.73	0.35–1.49	0.3850
[^18^F]FDG-PET vs. LP interval (mo)	**1.02**	**1.00–1.05**	**0.0473**	0.99	0.95–1.02	0.4534

	CSF AD biomarker results					
	*Consistent with AD vs. Not Consistent with AD/Equivocal*			*Not Consistent with AD vs. Consistent with AD/Equivocal*		
	OR	95% CI	*P*-value	OR	95% CI	*P*-value
PCG hypometabolism	**2.48**	**1.51–4.10**	**0.0003**	**0.21**	**0.08–0.47**	**0.0001**
Age (y)	0.99	0.97–1.02	0.6727	0.98	0.95–1.01	0.1191
Sex (Female as ref.)	0.69	0.45–1.07	0.0968	1.43	0.83–2.49	0.1971
[^18^F]FDG-PET vs. LP interval (mo)	**1.03**	**1.00–1.05**	**0.0140**	0.99	0.97–1.02	0.5669
	**OR**	**95% CI**	***P*-value**	**OR**	**95% CI**	***P*-value**

Frontal hypometabolism	1.21	0.78–1.87	0.3862	**0.41**	**0.23–0.72**	**0.0016**
Age (y)	0.99	0.97–1.02	0.5417	0.98	0.95–1.01	0.1379
Sex (Female as ref.)	0.73	0.47–1.13	0.1566	1.20	0.69–2.09	0.5185
[^18^F]FDG-PET vs. LP interval (mo)	**1.03**	**1.00–1.05**	**0.0135**	0.99	0.97–1.02	0.4629

[^18^F]FDG-PET vs. LP interval was treated as an interval variable, that is, as a continuous variable with negative values when the LP preceded the [^18^F]FDG-PET brain scan.

Statistically significant results are bold-faced.

Abbreviations: [^18^F]FDG = 2-[^18^F]fluoro-2-deoxy-d-glucose; AD = Alzheimer's disease; CI = confidence interval; CSF = cerebrospinal fluid; LP = lumbar puncture; OR = odds ratio; PCG = posterior cingulate gyrus; PET = Positron Emission Tomography.

We next evaluated whether these regional differences are also associated with the gold-standard CSF AD biomarker results. Indeed, PCG hypometabolism was significantly associated with a CSF Consistent with AD: individuals with PCG hypometabolism were more than twice as likely to have a CSF Consistent with AD (Table 5 and Fig. 4B) and ∼80% less likely to have a CSF profile Not Consistent with AD than individuals with spared or non-reported PCG metabolism, reinforcing the well-established specificity of PCG as an AD-vulnerable region^16^^,^^17^ (Table 5 and Fig. 4B). Again, in sharp contrast with the PCG, the presence of frontal hypometabolism was not significantly associated with a CSF Consistent with AD but subjects with frontal hypometabolism were ∼60% less likely to have a CSF Not Consistent with AD compared to those with spared or non-reported frontal metabolism, suggesting that the identification of frontal lobe hypometabolism may be misleading toward an imaging-based diagnosis of non-AD dementia (Table 5 and Fig. 4B).

Figure S1 illustrates the maps of Not AD-like, Inconclusive, and AD-like [^18^F]FDG-PET patterns split by CSF AD biomarker results. Tables S3–S7 depict the proportions of hypometabolism in each region for each [^18^F]FDG-PET and CSF AD biomarker group.

### Associations between clinical presentation, [^18^F]FDG-PET scan and CSF AD biomarker results, and final etiologic diagnosis

**Fig. 5:**
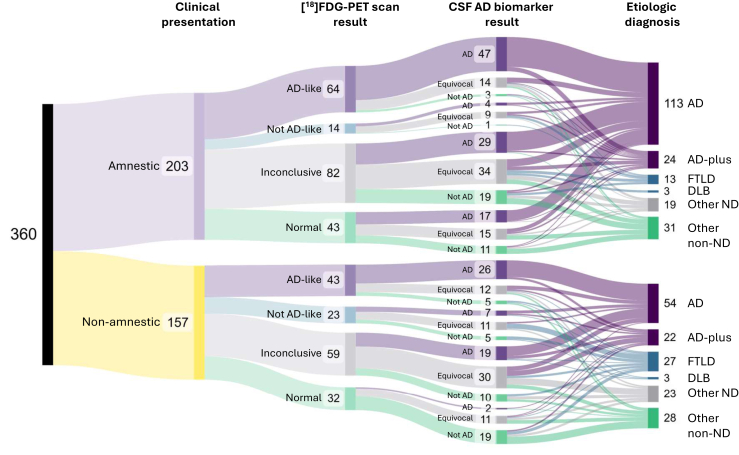
**Relationships between clinical presentation, [^18^F]FDG-PET brain scan results, CSF AD biomarker results, and the final etiologic diagnosis at last follow-up clinic visit.** Sankey diagram illustrating the path between clinical presentation and final etiologic diagnosis aided by [^18^F]FDG-PET brain scan and CSF AD biomarker panel. Flow widths are proportional to the number of subjects connecting the nodes. Abbreviations: AD = Alzheimer's disease; AD-plus = differential diagnosis between AD and another neurodegenerative or non-neurodegenerative cause or mixed AD and other neurodegenerative or non-neurodegenerative copathology; DLB = dementia with Lewy Bodies; FTLD = frontotemporal lobar degeneration; Other ND = other neurodegenerative disease (with or without another alternative or concurrent neurodegenerative or non-neurodegenerative diagnosis); Other non-ND = other non-neurodegenerative disease (e.g., vascular, normal pressure hydrocephalus, etc.).

We next investigated the associations between both clinical syndromic presentation and final etiologic diagnosis and the results of [^18^F]FDG-PET scan and CSF AD biomarker panel (Fig. 5). The Sankey diagram in Figure S2 shows the correlation between clinical presentation and final etiologic diagnosis without the [^18^F]FDG-PET scan and CSF AD biomarker panel results for a simpler visualization. As illustrated in the Sankey diagram in Fig. 5, regardless of the clinical syndromic presentation (amnestic or non-amnestic), virtually all patients with AD-like [^18^F]FDG-PET scan and CSF Consistent with AD received a final diagnosis of AD or AD-plus (in this case consisting of mixed AD and another neurodegenerative or non-neurodegenerative copathology). Moreover, expectedly, most patients (123/175, 70.3%) with Not AD-like, Inconclusive or Normal [^18^F]FDG-PET scan and Equivocal or Not Consistent with AD CSF biomarker panel received a final etiologic diagnosis other than AD, either another neurodegenerative disease (e.g., FTLD, DLB, limbic-predominant age-related TDP43 encephalopathy-neuropathologic change [LATE-NC], chronic traumatic encephalopathy [CTE], prion disease, Huntington's disease, etc.) or a non-neurodegenerative diagnosis (e.g., vascular dementia, normal-pressure hydrocephalus, anxiety/depression, substance abuse, normal aging).

On the other hand, when the [^18^F]FDG-PET scan was Inconclusive or read as Not AD-like or Normal, a CSF biomarker panel Consistent with AD trumped the [^18^F]FDG-PET results and led clinicians to diagnose their patients with AD or AD-plus in all but one patient in whom the predominant syndrome was progressive supranuclear palsy and the CSF results were dismissed by the neurologist. The final etiologic diagnoses of the eight patients with a false positive [^18^F]FDG-PET scan (i.e., CSF Not Consistent with AD but AD-like [^18^F]FDG-PET scan) included FTLD (three), DLB (one), mixed AD-DLB (one), vascular dementia (one), mild cognitive impairment secondary to alcohol use and mood disorders (one), and one patient was suspected to have normal-pressure hydrocephalus but was lost to follow up and died (no brain autopsy available). Of the 19 patients with a false negative [^18^F]FDG-PET brain scan (i.e., CSF Consistent with AD but Normal [^18^F]FDG-PET scan), 17 received a final diagnosis of AD or AD-plus (one mixed AD-DLB and another mixed AD-vascular), one was suspected to have prion disease due to a rapid course and a total Tau of 1740.4 pg/mL but the brain MRI and CSF RT-QuIC were not consistent with it, and another was found a concurrent anti-GAD65 autoimmune cerebellar syndrome with stiffness that overshadowed the clinical diagnosis of AD. Therefore, these data indicate that the clinicians prioritized the CSF AD biomarker panel results over the [^18^F]FDG-PET scan results whenever they were discrepant.

## Discussion

In this real-world tertiary memory clinic cohort, [^18^F]FDG-PET demonstrated high specificity but limited sensitivity to identify the AD pathophysiological process as defined by CSF biomarkers. While an AD-like [^18^F]FDG-PET pattern independently predicted a CSF Consistent with AD, the agreement between [^18^F]FDG-PET brain scan and CSF AD biomarkers was only fair and the small positive and negative likelihood ratios indicated that the [^18^F]FDG-PET brain scan narrative results (AD-like vs. other) have only a modest association with the probability of a positive CSF AD biomarker panel. We found 12.6% false negative and 11.0% false positive results of the [^18^F]FDG-PET brain scan when compared with CSF AD biomarkers. [^18^F]FDG-PET imaging is considered a broad biomarker of neurodegeneration but, in the context of AD, [^18^F]FDG-PET hypometabolism tracks well with the severity of local cortical atrophy as measured with structural MRI and with the extent and distribution of tau neurofibrillary tangles as assessed with tau PET imaging.^19^ Therefore, false negative [^18^F]FDG-PET results may represent an earlier disease stage in which tau-induced neurodegeneration is not yet widespread.^20^ On the other hand, false positive [^18^F]FDG-PET results (i.e., discordant cases with AD-like [^18^F]FDG-PET, but CSF Not Consistent with AD) were likely driven by non-AD neuropathologies mimicking the classic regional hypometabolism of AD, as judged by the final diagnoses adjudicated to those patients by expert behavioral neurologists.

As expected, the regional pattern of hypometabolism in the [^18^F]FDG-PET scans determined the likelihood of a report of AD-like vs. other results, but notably also correlated with the CSF AD biomarker panel results. In particular, the reporting of PCG hypometabolism was not only strongly associated with an AD-like [^18^F]FDG-PET result but also with a CSF Consistent with AD, thus supporting a correct etiological diagnosis. This finding also underscores the adherence of our center's nuclear medicine specialists to existing practice guidelines^7^^,^^8^ and reinforces the importance of evaluating PCG glucose metabolism to discern between AD and non-AD dementias in a memory clinic setting.^7^^,^^8^ Conversely, there was a dissociation between the [^18^F]FDG-PET and CSF correlates of a frontal hypometabolism report. On one hand, frontal hypometabolism was strongly associated with a Not AD-like pattern of [^18^F]FDG-PET results, suggesting that scan readers substantially rely on this region to adjudicate their final etiological diagnosis; on the other hand, individuals with frontal hypometabolism were ∼60% less likely to have a CSF Not Consistent with AD (vs. Consistent with AD/Equivocal) than those with normal or non-reported frontal metabolism. Thus, the finding of frontal hypometabolism in the [^18^F]FDG-PET scan may have misled readers toward a non-AD etiology but based on these data, unlike PCG, frontal hypometabolism cannot be used to confidently predict the underlying pathology.

Previous studies investigating the performance of [^18^F]FDG-PET against CSF AD biomarkers have reported kappa coefficients consistent with moderate or substantial agreement.^10^, ^11^, ^12^, ^13^ Several methodological differences between those studies and ours (reviewed in Table 6) could explain these disparate conclusions including differences in sample size, number of specialists reading the [^18^F]FDG-PET images and, perhaps, patient complexity, since our center offers specialized clinics in FTLD, DLB, and vascular dementia in addition to general memory clinics.

**Table 6:** Studies comparing [^18^F]FDG-PET imaging with CSF AD biomarkers in academic tertiary memory clinics.

Author (year)	City (Country)	Sample size	Number of [^18^F]FDG-PET readers	CSF-based AD prevalence (%)	PPV (95% CI)	NPV (95% CI)	[^18^F]FDG-PET vs. CSF AD biomarkers κ coefficient (95% CI)
Rubí et al. (2018)	Palma de Mallorca (Spain)	120	1	17.5 (21/120)	0.34 (0.21–0.50)	0.90 (0.82–0.95)	0.46 (0.35–0.57)
Quispialaya et al. (2022)	Montreal (Canada)	136	2	38.2 (52/136)	0.72 (0.60–0.82)	0.87 (0.78–0.93)	0.60 (0.46–0.74)
Mimenza-Alvarado et al. (2024)	Mexico City (Mexico)	25	2	32.0 (8/25)	0.78 (0.45–0.96)	0.94 (0.72–1.00)	0.733 (0.452–1.000)
Quispialaya et al. (2025)	Montreal (Canada)	81	2	42.0 (34/81)	0.64 (0.48–0.77)	0.79 (0.64–0.88)	0.43 (0.23–0.62)
Rabaneda-Lombarte et al. (2026)	Boston (USA)	360	28	41.9 (151/360)	0.68 (0.59–0.76)	0.69 (0.63–0.75)	0.334 (0.237–0.431)

PPV and NPV were calculated based on study prevalence of CSF-defined AD.

Abbreviations: [^18^F]FDG = 2-[^18^F]fluoro-2-deoxy-d-glucose; AD = Alzheimer's disease; CI = confidence interval; CSF = cerebrospinal fluid; NPV = negative predictive value; PET = Positron Emission Tomography; PPV = positive predictive value.

Our study has limitations inherent to its real-world design. First, to mimic the real-world interpretation of [^18^F]FDG-PET scan reports by ordering clinicians, we relied exclusively on the narrative reports for the categorization of [^18^F]FDG-PET results; using global and regional SUVRs and new promising AI-based methods^21^^,^^22^ might have rendered a higher diagnostic accuracy of [^18^F]FDG-PET imaging but these approaches are less accessible to the clinician. Second, the inconsistent order and time interval between [^18^F]FDG-PET scan and lumbar puncture may have influenced [^18^F]FDG-PET reporting practices and affected the concordance between [^18^F]FDG-PET and CSF AD biomarker results; indeed, our sensitivity analyses pointed to some degree of information bias when the lumbar puncture preceded the [^18^F]FDG-PET scan, however we tried to account for this in our regression analyses by treating the time between both tests as an interval variable (i.e., a continuous variable with negative values when the lumbar puncture preceded the [^18^F]FDG-PET scan). Third, aging-related co-pathologies not accounted for in this study are commonly found in patients in this specialized clinical setting and can modify the [^18^F]FDG-PET hypometabolism pattern,^23^, ^24^, ^25^ thereby contributing to reporting uncertainty and limited concordance with CSF AD biomarkers. Fourth, we could not account for variability in the experience of [^18^F]FDG-PET scan readers.

Strengths include the large sample of patients from a tertiary referral center with expertise in AD and related dementias such as DLB and FTLDs; the real-world study design with a systematic collection and categorization of [^18^F]FDG-PET scan results solely based on the nuclear medicine department narrative reports, with adjudication blinded to the CSF AD biomarker results; and the analyses highlighting the reporting practices of [^18^F]FDG-PET scan results by nuclear medicine specialists in this clinical setting.

In summary, in this academic tertiary referral memory clinic setting, [^18^F]FDG-PET imaging demonstrated high specificity but limited sensitivity to identify AD as defined by CSF biomarker criteria. Although a typical AD-like [^18^F]FDG-PET pattern of hypometabolism predicted a positive CSF AD biomarker panel, the agreement between [^18^F]FDG-PET and CSF AD biomarker results was only fair. These findings imply that [^18^F]FDG-PET and CSF AD biomarkers are complementary rather than interchangeable diagnostic tests and that clinicians should integrate their results with all other available clinical information to refine their differential diagnosis and therapeutic management. Moreover, these findings should encourage clinicians to pursue AD biomarker testing even if the [^18^F]FDG-PET scan is inconclusive or not AD-like, particularly if the clinical suspicion for AD remains high.

## Contributors

NR-L: Data curation, Formal analysis, Funding acquisition, Investigation, Visualization, Writing – original draft, Writing – review & editing; J-PFS: Software, Visualization, Writing – review & editing; JC: Data curation; ERZ: Resources, Writing – review & editing; BCD: Writing – review & editing; SEA: Resources, Writing – review & editing; PK: Data curation, Resources, Writing – review & editing; AS-P: Conceptualization, Formal analysis, Funding acquisition, Methodology, Project administration, Supervision, Validation, Writing – original draft, Writing – review & editing. All authors had full access to all data in the study and have full responsibility for the decision to submit to publication. NR-L and AS-P had access and verified the data.

## Data sharing statement

Deidentified data are available upon reasonable request to the corresponding author (aserrano1@mgh.harvard.edu) and Mass General Brigham Institutional Review Board approval.

## Declaration of interests

NR-L received a research fellowship from Fundación Ramón Areces. ERZ has received research grants from the Alzheimer's Association, CAPES, Ciencia Pioneira, CNPq, FAPERGS, the Michael J. Fox Foundation, National Academy of Neuropsychology, and Serrapilheira; consulting fees from Biogen, Nintx, Novo Nordisk, and Magdalena Biosciences; payment or honoraria for lectures, presentations, speakers bureaus from Biogen, Lilly and Novo Nordisk; and support for attending meetings and/or travel from the Alzheimer's Association, Biogen, Capes, CNPq, and Novo Nordisk. He is also a co-founder and minority shareholder of MASIMA. BDC has received royalties from Oxford University Press and Cambridge University Press, and consulting fees from Biogen, Cervomed, Eisai, Lantheus, Lilly, Merck, Novo Nordisk, and Quanterix. SEA has received research funds from AbbVie, AC Immune, Alzheimer's Association, Athira, Challenger Foundation, Chromadex, Eli Lilly, Fortrea, Gatehouse Bio, Ionis Pharmaceuticals, Janssen Pharmaceuticals, John Sperling Foundation, National Institute of Health, Novartis, Seer Biosciences, Superfluid Dx, and Venture Well; consulting fees from Allyx Therapeutics, BioVie, Bob's Last Marathon, Jocasta Neuroscience, Merck, Sage Therapeutics, Sanofi, and Vandria; and expert witness fees from Foster & Eldredge and ProSelect Insurance Co. AS-P declares a material transfer agreement with Ionis Pharmaceuticals and research funds from AbbVie. JPF-S, JC, and PK declare no competing interest related to this article content.
