# Supplementary material for: Real-world comparison of brain [18F]FDG-PET imaging with CSF Alzheimer's disease biomarkers in a tertiary memory clinic setting
Source: eClinicalMedicine. 2026 Apr 20;95:103910. doi: 10.1016/j.eclinm.2026.103910 (PMC13121422; doi:10.1016/j.eclinm.2026.103910)
Supplement: Tables and Figures [file mmc1.pdf]

SUPPLEMENTAL TABLES

Table S1. Contingency tables and performance of [^{18}F]FDG-PET relative to CSF AD biomarker results split by CSF biomarker assay.

Athena ADmark™ (n=311)					
		**[^{18}F]FDG-PET**			Sensitivity: 0.49 (0.40 – 0.57)<br>Specificity: 0.83 (0.77 – 0.88)<br>PPV: 0.67 (0.57 – 0.76)<br>NPV: 0.69 (0.63 – 0.75)<br>LR+: 2.837; LR-: 0.617<br>AUC: 0.658 (0.607 – 0.709)<br>Agreement: 68.5%<br>Kappa: 0.329 (0.224 – 0.433)
		**AD-like**	**Other**		
**CSF**	**AD**	64 (67.4)	67 (31.0)	131	
	**Other**	31 (32.6)	149 (69.0)	180	
		95	216	311	

Roche Elecsys™ (n=49)					
		**[^{18}F]FDG-PET**			Sensitivity: 0.45 (0.25 – 0.65)<br>Specificity: 0.90 (0.74 – 0.96)<br>PPV: 0.75 (0.47 – 0.91)<br>NPV: 0.70 (0.54 – 0.83)<br>LR+: 4.350; LR-: 0.613<br>AUC: 0.673 (0.548 – 0.799)<br>Agreement: 71.4%<br>Kappa: 0.369 (0.114 – 0.625)
		**AD-like**	**Other**		
**CSF**	**AD**	9 (75.0)	11 (29.7)	20	
	**Other**	3 (25.0)	26 (70.3)	29	
		12	37	49	

Ranges in parentheses represent the 95% confidence intervals. Abbreviations: AD = Alzheimer's disease; AUC = area under the curve of the receiver operating characteristic; CSF = cerebrospinal fluid; [^{18}F]FDG = 2-[^{18}F]fluoro-2-deoxy-D-glucose; LP = lumbar puncture; LR = likelihood ratio; NPV = negative predictive value; PET = Positron Emission Tomography; PPV = positive predictive value.

Table S2. Frequency of hypometabolism across brain regions by [^{18}F]FDG-PET laterality/symmetry groups.

Brain region	Bilateral symmetric (n = 100)	Bilateral L > R (n = 85)	Bilateral R > L (n = 66)	L Unilateral (n = 24)	R Unilateral (n = 9)	Overall (n = 285)
Anterior cingulate L	9 (9.0)	6 (7.1)	8 (12.1)	2 (8.3)	0 (0.0)	25 (8.8)
Anterior cingulate R	9 (9.0)	6 (7.1)	8 (12.1)	0 (0.0)	0 (0.0)	23 (8.1)
Basal ganglia L	4 (4.0)	8 (9.4)	3 (4.5)	3 (12.5)	0 (0.0)	18 (6.3)
Basal ganglia R	4 (4.0)	5 (5.9)	5 (7.6)	1 (4.2)	1 (11.1)	16 (5.6)
Frontal L	47 (47.0)	56 (65.9)	39 (59.1)	8 (33.3)	0 (0.0)	150 (52.6)
Frontal R	47 (47.0)	44 (51.8)	46 (69.7)	0 (0.0)	5 (55.6)	142 (49.8)
Insula L	0 (0.0)	1 (1.2)	0 (0.0)	2 (8.3)	0 (0.0)	3 (1.1)
Insula R	0 (0.0)	1 (1.2)	0 (0.0)	0 (0.0)	0 (0.0)	1 (0.4)
Occipital L	14 (14.0)	15 (17.6)	14 (21.2)	6 (25.0)	1 (11.1)	50 (17.5)
Occipital R	14 (14.0)	10 (11.8)	16 (24.2)	0 (0.0)	1 (11.1)	41 (14.4)
Parietal L	65 (65.0)	76 (89.4)	54 (81.8)	15 (62.5)	0 (0.0)	210 (73.7)
Parietal R	65 (65.0)	70 (82.4)	57 (86.4)	0 (0.0)	8 (88.9)	200 (70.2)
Posterior cingulate L	25 (25.0)	33 (38.8)	22 (33.3)	3 (12.5)	0 (0.0)	83 (29.1)
Posterior cingulate R	25 (25.0)	26 (30.6)	27 (40.9)	0 (0.0)	0 (0.0)	78 (27.4)
Precuneus L	19 (19.0)	18 (21.2)	21 (31.8)	2 (8.3)	0 (0.0)	60 (21.1)
Precuneus R	19 (19.0)	12 (14.1)	23 (34.8)	0 (0.0)	0 (0.0)	54 (18.9)
Temporal L	70 (70.0)	76 (89.4)	56 (84.8)	17 (70.8)	0 (0.0)	219 (76.8)
Temporal R	70 (70.0)	62 (72.9)	59 (89.4)	0 (0.0)	6 (66.7)	197 (69.1)

Data are presented as n (%) of individuals with hypometabolism for each brain region across groups. Note: participants with normal [^{18}F]FDG-PET were not included in this analysis. Abbreviations: [^{18}F]FDG = 2-[^{18}F]fluoro-2-deoxy-D-glucose; L = left; PET = Positron Emission Tomography; R = right.

Table S3. Frequency of hypometabolism across brain regions by [^{18}F]FDG-PET groups.

Brain region	Not AD-like (n = 37)	Inconclusive (n = 141)	AD-like (n = 107)	Overall (n = 285)
Anterior cingulate L	5 (13.5)	16 (11.3)	4 (3.7)	25 (8.8)
Anterior cingulate R	4 (10.8)	16 (11.3)	3 (2.8)	23 (8.1)
Basal ganglia L	7 (18.9)	10 (7.1)	1 (0.9)	18 (6.3)
Basal ganglia R	7 (18.9)	8 (5.7)	1 (0.9)	16 (5.6)
Frontal L	30 (81.1)	72 (51.1)	48 (44.9)	150 (52.6)
Frontal R	26 (70.3)	71 (50.4)	45 (42.1)	142 (49.8)
Insula L	2 (5.4)	1 (0.7)	0 (0.0)	3 (1.1)
Insula R	0 (0.0)	1 (0.7)	0 (0.0)	1 (0.4)
Occipital L	6 (16.2)	29 (20.6)	15 (14.0)	50 (17.5)
Occipital R	3 (8.1)	24 (17.0)	14 (13.1)	41 (14.4)
Parietal L	20 (54.1)	91 (64.5)	99 (92.5)	210 (73.7)
Parietal R	19 (51.4)	88 (62.4)	93 (86.9)	200 (70.2)
Posterior cingulate L	2 (5.4)	30 (21.3)	51 (47.7)	83 (29.1)
Posterior cingulate R	2 (5.4)	30 (21.3)	46 (43.0)	78 (27.4)
Precuneus L	2 (5.4)	25 (17.7)	33 (30.8)	60 (21.1)
Precuneus R	2 (5.4)	25 (17.7)	27 (25.2)	54 (18.9)
Temporal L	28 (75.7)	94 (66.7)	97 (90.7)	219 (76.8)
Temporal R	23 (62.2)	87 (61.7)	87 (81.3)	197 (69.1)

Data are presented as n (%) of individuals with hypometabolism for each brain region across groups. Note: participants with normal [^{18}F]FDG-PET were not included in this analysis. Abbreviations: [^{18}F]FDG = 2-[^{18}F]fluoro-2-deoxy-D-glucose; L = left; PET = Positron Emission Tomography; R = right.

Table S4. Frequency of hypometabolism across brain regions by CSF AD biomarker groups.

Brain region	Not Consistent with AD (n = 43)	Equivocal (n = 110)	Consistent with AD (n = 132)	Overall (n = 285)
Anterior cingulate L	4 (9.3)	11 (10.0)	10 (7.6)	25 (8.8)
Anterior cingulate R	4 (9.3)	9 (8.2)	10 (7.6)	23 (8.1)
Basal ganglia L	4 (9.3)	10 (9.1)	4 (3.0)	18 (6.3)
Basal ganglia R	3 (7.0)	10 (9.1)	3 (2.3)	16 (5.6)
Frontal L	19 (44.2)	67 (60.9)	64 (48.5)	150 (52.6)
Frontal R	16 (37.2)	60 (54.5)	66 (50.0)	142 (49.8)
Insula L	0 (0.0)	3 (2.7)	0 (0.0)	3 (1.1)
Insula R	0 (0.0)	1 (0.9)	0 (0.0)	1 (0.4)
Occipital L	4 (9.3)	17 (15.5)	29 (22.0)	50 (17.5)
Occipital R	2 (4.7)	14 (12.7)	25 (18.9)	41 (14.4)
Parietal L	22 (51.2)	72 (65.5)	116 (87.9)	210 (73.7)
Parietal R	19 (44.2)	69 (62.7)	112 (84.8)	200 (70.2)
Posterior cingulate L	6 (14.0)	30 (27.3)	47 (35.6)	83 (29.1)
Posterior cingulate R	4 (9.3)	28 (25.5)	46 (34.8)	78 (27.4)
Precuneus L	6 (14.0)	15 (13.6)	39 (29.5)	60 (21.1)
Precuneus R	5 (11.6)	13 (11.8)	36 (27.3)	54 (18.9)
Temporal L	29 (67.4)	78 (70.9)	112 (84.8)	219 (76.8)
Temporal R	23 (53.5)	69 (62.7)	105 (79.5)	197 (69.1)

Data are presented as n (%) of individuals with hypometabolism for each brain region across groups. Note: participants with normal [^{18}F]FDG-PET were not included in this analysis. Abbreviations: [^{18}F]FDG = 2-[^{18}F]fluoro-2-deoxy-D-glucose; L = left; PET = Positron Emission Tomography; R = right.

Table S5. Frequency of hypometabolism across brain regions by CSF AD biomarker groups in the Abnormal Not AD-like [^{18}F]FDG-PET subgroup.

Brain region	Not Consistent with AD (n = 6)	Equivocal (n = 20)	Consistent with AD (n = 11)	Overall (n = 37)
Anterior cingulate L	1 (16.7)	4 (20.0)	0 (0.0)	5 (13.5)
Anterior cingulate R	1 (16.7)	3 (15.0)	0 (0.0)	4 (10.8)
Basal ganglia L	2 (33.3)	3 (15.0)	2 (18.2)	7 (18.9)
Basal ganglia R	1 (16.7)	4 (20.0)	2 (18.2)	7 (18.9)
Frontal L	4 (66.7)	18 (90.0)	8 (72.7)	30 (81.1)
Frontal R	3 (50.0)	15 (75.0)	8 (72.7)	26 (70.3)
Insula L	0 (0.0)	2 (10.0)	0 (0.0)	2 (5.4)
Insula R	0 (0.0)	0 (0.0)	0 (0.0)	0 (0.0)
Occipital L	2 (33.3)	1 (5.0)	3 (27.3)	6 (16.2)
Occipital R	1 (16.7)	1 (5.0)	1 (9.1)	3 (8.1)
Parietal L	4 (66.7)	8 (40.0)	8 (72.7)	20 (54.1)
Parietal R	3 (50.0)	9 (45.0)	7 (63.6)	19 (51.4)
Posterior cingulate L	0 (0.0)	2 (10.0)	0 (0.0)	2 (5.4)
Posterior cingulate R	0 (0.0)	2 (10.0)	0 (0.0)	2 (5.4)
Precuneus L	0 (0.0)	0 (0.0)	2 (18.2)	2 (5.4)
Precuneus R	0 (0.0)	0 (0.0)	2 (18.2)	2 (5.4)
Temporal L	5 (83.3)	15 (75.0)	8 (72.7)	28 (75.7)
Temporal R	4 (66.7)	12 (60.0)	7 (63.6)	23 (62.2)

Data are presented as n (%) of individuals with hypometabolism for each brain region across groups. Abbreviations: AD = Alzheimer’s disease; CSF= cerebrospinal fluid; [^{18}F]FDG = 2-[^{18}F]fluoro-2-deoxy-D-glucose; L = left; PET = Positron Emission Tomography; R = right.

Table S6. Frequency of hypometabolism across brain regions by CSF AD biomarker groups in the Abnormal Inconclusive [^{18}F]FDG-PET subgroup.

Brain region	Not Consistent with AD (n = 29)	Equivocal (n = 64)	Consistent with AD (n = 48)	Overall (n = 141)
Anterior cingulate L	3 (10.3)	7 (10.9)	6 (12.5)	16 (11.3)
Anterior cingulate R	3 (10.3)	6 (9.4)	7 (14.6)	16 (11.3)
Basal ganglia L	2 (6.9)	6 (9.4)	2 (4.2)	10 (7.1)
Basal ganglia R	2 (6.9)	5 (7.8)	1 (2.1)	8 (5.7)
Frontal L	11 (37.9)	34 (53.1)	27 (56.2)	72 (51.1)
Frontal R	10 (34.5)	32 (50.0)	29 (60.4)	71 (50.4)
Insula L	0 (0.0)	1 (1.6)	0 (0.0)	1 (0.7)
Insula R	0 (0.0)	1 (1.6)	0 (0.0)	1 (0.7)
Occipital L	2 (6.9)	14 (21.9)	13 (27.1)	29 (20.6)
Occipital R	1 (3.4)	11 (17.2)	12 (25.0)	24 (17.0)
Parietal L	10 (34.5)	41 (64.1)	40 (83.3)	91 (64.5)
Parietal R	9 (31.0)	38 (59.4)	41 (85.4)	88 (62.4)
Posterior cingulate L	3 (10.3)	15 (23.4)	12 (25.0)	30 (21.3)
Posterior cingulate R	3 (10.3)	15 (23.4)	12 (25.0)	30 (21.3)
Precuneus L	2 (6.9)	10 (15.6)	13 (27.1)	25 (17.7)
Precuneus R	2 (6.9)	8 (12.5)	15 (31.2)	25 (17.7)
Temporal L	17 (58.6)	40 (62.5)	37 (77.1)	94 (66.7)
Temporal R	14 (48.3)	36 (56.2)	37 (77.1)	87 (61.7)

Data are presented as n (%) of individuals with hypometabolism for each brain region across groups. Abbreviations: AD = Alzheimer's disease; CSF = cerebrospinal fluid; [^{18}F]FDG = 2-[^{18}F]fluoro-2-deoxy-D-glucose; L = left; PET = Positron Emission Tomography; R = right.

Table S7. Frequency of hypometabolism across brain regions by CSF AD biomarker groups in the Abnormal AD-like [18F]FDG-PET subgroup.

Brain region	Not Consistent with AD (n = 8)	Equivocal (n = 26)	Consistent with AD (n = 73)	Overall (n = 107)
Anterior cingulate L	0 (0.0)	0 (0.0)	4 (5.5)	4 (3.7)
Anterior cingulate R	0 (0.0)	0 (0.0)	3 (4.1)	3 (2.8)
Basal ganglia L	0 (0.0)	1 (3.8)	0 (0.0)	1 (0.9)
Basal ganglia R	0 (0.0)	1 (3.8)	0 (0.0)	1 (0.9)
Frontal L	4 (50.0)	15 (57.7)	29 (39.7)	48 (44.9)
Frontal R	3 (37.5)	13 (50.0)	29 (39.7)	45 (42.1)
Insula L	0 (0.0)	0 (0.0)	0 (0.0)	0 (0.0)
Insula R	0 (0.0)	0 (0.0)	0 (0.0)	0 (0.0)
Occipital L	0 (0.0)	2 (7.7)	13 (17.8)	15 (14.0)
Occipital R	0 (0.0)	2 (7.7)	12 (16.4)	14 (13.1)
Parietal L	8 (100.0)	23 (88.5)	68 (93.2)	99 (92.5)
Parietal R	7 (87.5)	22 (84.6)	64 (87.7)	93 (86.9)
Posterior cingulate L	3 (37.5)	13 (50.0)	35 (47.9)	51 (47.7)
Posterior cingulate R	1 (12.5)	11 (42.3)	34 (46.6)	46 (43.0)
Precuneus L	4 (50.0)	5 (19.2)	24 (32.9)	33 (30.8)
Precuneus R	3 (37.5)	5 (19.2)	19 (26.0)	27 (25.2)
Temporal L	7 (87.5)	23 (88.5)	67 (91.8)	97 (90.7)
Temporal R	5 (62.5)	21 (80.8)	61 (83.6)	87 (81.3)

Data are presented as n (%) of individuals with hypometabolism for each brain region across groups. Abbreviations: AD = Alzheimer's disease; CSF = cerebrospinal fluid; [18F]FDG = 2-[18F]fluoro-2-deoxy-D-glucose; L = left; PET = Positron Emission Tomography; R = right.

SUPPLEMENTAL FIGURES

Figure S1

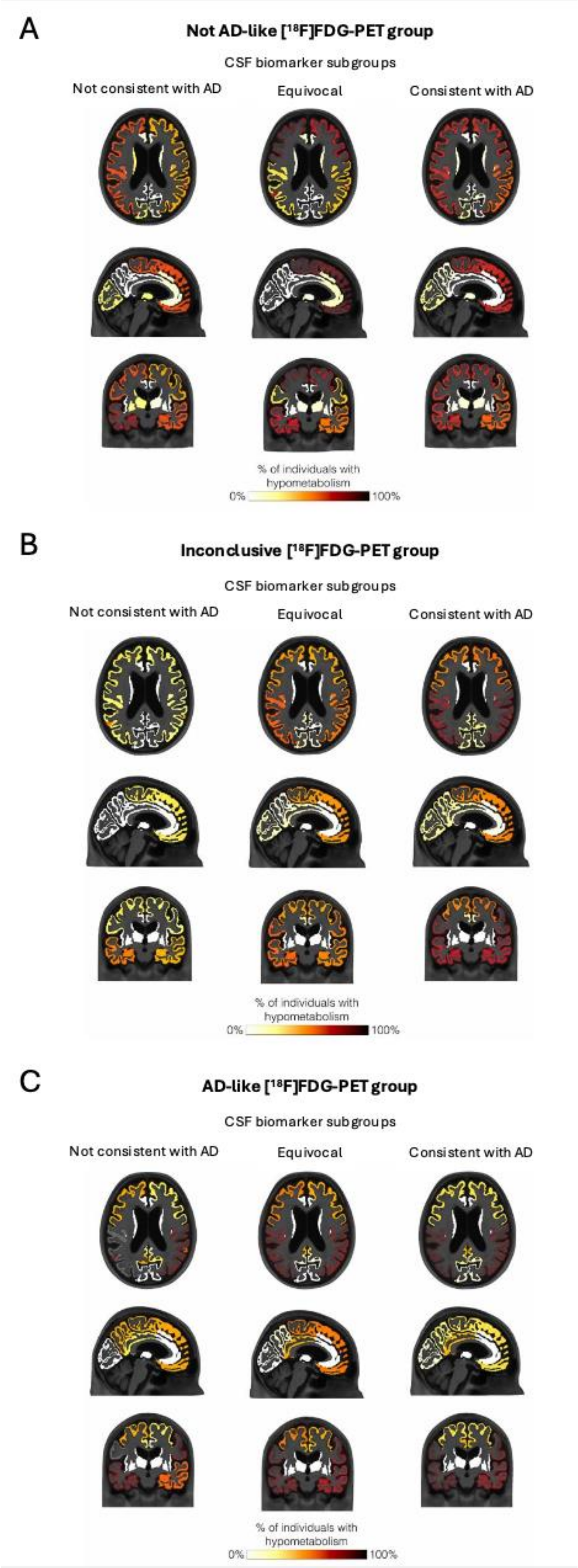

Figure S1. A report of posterior cingulate gyrus hypometabolism correlates with a CSF Consistent with AD across [^{18}F]FDG-PET patterns. Mapping of proportions of individuals with each hypometabolic area by CSF AD biomarker result categories for the (**A**) Abnormal Not AD-like, (**B**) Abnormal AD-like, and (**C**) Abnormal Inconclusive [^{18}F]FDG-PET categories. Abbreviations: AD = Alzheimer's disease; CSF = cerebrospinal fluid; [^{18}F]FDG = 2-[18F]fluoro-2-deoxy-D-glucose; L = left; PET = Positron Emission Tomography; R = right.

Figure S2

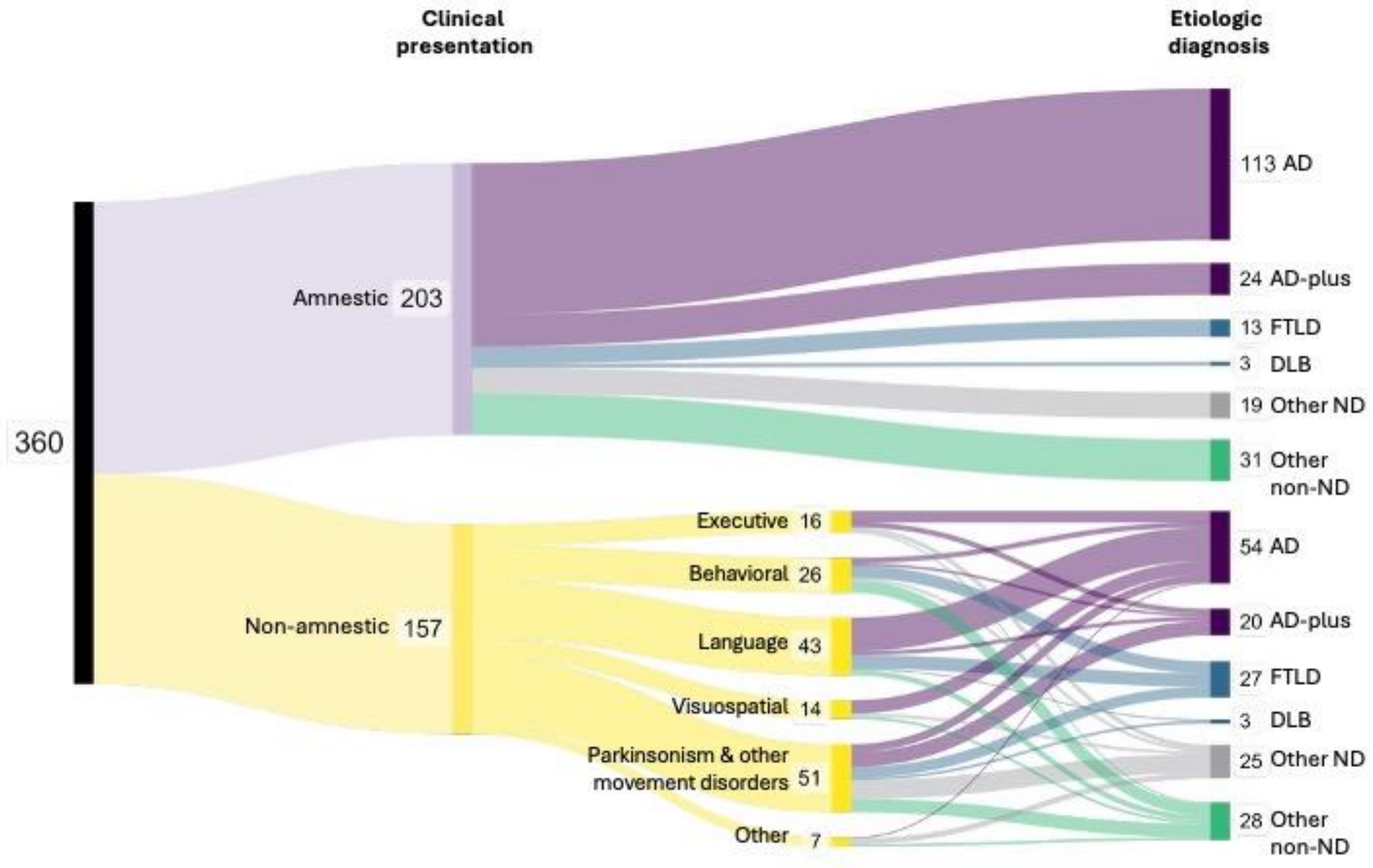

Clinical presentation
Etiologic diagnosis
360
Amnestic 203
Non-amnestic 157
Executive 16
Behavioral 26
Language 43
Visuospatial 14
Parkinsonism & other movement disorders 51
Other 7
113 AD
24 AD-plus
13 FTLD
3 DLB
19 Other ND
31 Other non-ND
54 AD
20 AD-plus
27 FTLD
3 DLB
25 Other ND
28 Other non-ND

Figure S2. Relationship between clinical presentation and etiologic diagnosis at last follow-up clinic visit. Sankey diagram illustrates the path from the specific clinical syndromic presentation based on the evaluation at the first clinic visit and the final etiologic diagnosis at last follow-up clinic visit after [^{18}F]FDG-PET brain scan and CSF AD biomarkers. Flow widths are proportional to the number of subjects connecting the nodes. Abbreviations: AD = Alzheimer's disease; AD-plus = differential diagnosis between AD and another neurodegenerative or non-neurodegenerative cause or mixed AD and other neurodegenerative or non-neurodegenerative copathology; DLB = dementia with Lewy Bodies; FTLD = frontotemporal lobar degeneration; Other ND = other neurodegenerative disease (with or without another alternative or concurrent neurodegenerative or non-neurodegenerative diagnosis); Other non-ND = other non-neurodegenerative disease (e.g., vascular, normal pressure hydrocephalus, etc.).
